# Supplementary material for: metaGE: Investigating genotype x environment interactions through GWAS meta-analysis
Source: PLoS Genet. 2025 Jan 10;21(1):e1011553. doi: 10.1371/journal.pgen.1011553 (PMC11756807; doi:10.1371/journal.pgen.1011553)
Supplement: S1 Text — (PDF) [file pgen.1011553.s001.pdf]

Supporting Information for

metaGE: Investigating genotype x environment interactions through GWAS meta-analysis

Annaïg De Walsche, Alexis Vergne, Renaud Rincent, Fabrice Roux, Stéphane Nicolas, Claude Welcker, Sofiane Mezmouk, Alain Charcosset and Tristan Mary-Huard

Corresponding Author name: Tristan Mary-Huard.
E-mail: tristan.mary-huard@agroparistech.fr

Supporting Information Text 1: Meta-analysis classical approach

In this section, we present the fixed effect (1) and the random effect (2, 3) meta-analyses that are traditionally used in human genetics.

Fixed effect procedure.

Model We consider a meta-analysis relying on K different genetic association studies testing the association between a set of M markers and a phenotype of interest. We designate β_{mk} as the estimated effect of marker m in study k, and p_{mk} as its associated p-value. We define the z-scores Z_{mk} as

$$Z_{mk} = -\Phi^{-1}(0.5 p_{mk}) \times \text{sign}(\beta_{mk}),$$

where Φ^{-1} stands for the standard Gaussian cumulative distribution function. The z-score is to be understood as follows: the smaller the p-value of the marker, the greater the absolute value of the z-score, with the sign of the z-score corresponding to the sign of the marker effect. Importantly, when marker m is not associated with the phenotype, Z_{mk} follows a standard Gaussian distribution, *i.e.* the H_0 distribution of Z_{mk} is known. The fixed-effect model assumes the effect of a marker to be stable (*i.e.* identical) across studies. Designating $Z_m := (Z_{m1}, \ldots, Z_{mK})$ as the vector of z-scores of marker m, one has:

$$\begin{aligned} Z_m &= \mu_m \mathbb{1}_K + E_m \\ E_m &\sim \mathcal{N}(0_K, I) \end{aligned}$$

where $\mu_m \in \mathbb{R}$ quantifies the deviation to H_0 of marker m and is common to all studies, E_m is the vector of error terms, and I is the identity matrix. Note that the model assumes the z-scores to be mutually independent. This assumption is satisfied whenever the initial GWAS analyses are performed on different panels, a classical configuration in human genetics where MA is usually performed to summarize GWAS performed on different populations.

Inference The parameter μ_m can be easily inferred using the empirical mean of Z_m :

$$\hat{\mu}_m = \frac{1}{K} \sum_{k=1}^{K} Z_{mk}$$

One can then perform association detection by testing

$$H_0 : \{\mu_m = 0\} \quad \text{vs} \quad H_1 : \{\mu_m \neq 0\}$$

based on the following test statistic

$$S_m = \sqrt{K} \hat{\mu}_m = \frac{1}{\sqrt{K}} \sum_{k=1}^{K} Z_{mk}$$

that follows a $\mathcal{N}(0, 1)$ distribution under the null hypothesis, which corresponds to the approach of the `METAL` procedure (4). The resulting MA p-value for marker m is then

$$p_m = 2 \times \Phi\left(-|s_m|\right),$$

with s_m the observed value of S_m.

Random effect procedure.

Model We designate β_{mk} as the estimated effect of marker m in study k, and v_{mk} its associated standard error. The random-effect model incorporates the heterogeneity of the marker effects across studies. Designating $\beta_m := (\beta_{m1}, \ldots, \beta_{mK})$ as the vector of the estimated effect of marker m, one has:

$$\begin{aligned} \beta_m &= \mu_m \mathbb{1}_K + A_m + E_m, \\ A_m &\sim \mathcal{N}(0, \tau_m^2 I) \\ E_m &\sim \mathcal{N}(0, V_m) \\ A_m &\perp\!\!\!\perp E_m \end{aligned}$$

where $\mu_m \in \mathbb{R}$ quantifies the deviation to H_0 of marker m and is common to all studies, τ_m is the between-study variance associated with the random marker effect A_m, E_m is the vector of error terms, V_m is the diagonal matrix of $(v_{mk}^2)_{k=1,\ldots,K}$ and I is the identity matrix.

Inference The maximum likelihood estimators of μ_m and τ_m^2 are obtained by solving the following equations iteratively (5):

$$\hat{\mu}_m = \frac{\sum_{k=1}^{K} \frac{\beta_{mk}}{(v_{mk}^2 + \hat{\tau}_m^2)}}{\sum_{k=1}^{K} \frac{1}{(v_{mk}^2 + \hat{\tau}_m^2)}}$$

$$\hat{\tau}_m^2 = \frac{\sum_{k=1}^{K} \frac{(\beta_{mk} - \hat{\mu}_m)^2 - v_{mk}^2}{(v_{mk}^2 + \hat{\tau}_m^2)^2}}{\sum_{k=1}^{K} \frac{1}{(v_{mk}^2 + \hat{\tau}_m^2)^2}}$$

The test of the association of the marker corresponds to:

$$H_0 : \{\mu_m = 0 \text{ and } \tau_m^2 = 0\} \quad \text{vs} \quad H_1 : \{\mu_m \neq 0 \text{ or } \tau_m^2 \neq 0\}$$

and can be performed using a likelihood ratio test (3). Designating l_0 and l_1 as the likelihood of β_m under H_0 and H_1, respectively, the test statistic is:

$$2(l_1 - l_0) \overset{H_0}{\sim} \frac{1}{2}\chi^2(1) + \frac{1}{2}\chi^2(2)$$

References

1. A Whitehead, J Whitehead, A general parametric approach to the meta-analysis of randomized clinical trials. *Stat. medicine* **10**, 1665–1677 (1991).
2. R DerSimonian, N Laird, Meta-analysis in clinical trials. *Control. Clin. Trials* **7**, 177–188 (1986).
3. B Han, E Eskin, Random-effects model aimed at discovering associations in meta-analysis of genome-wide association studies. *The Am. J. Hum. Genet.* (2011).
4. CJ Willer, Y Li, GR Abecasis, Metal: Fast and efficient meta-analysis of genomewide association scans. *Bioinformatics* **26**, 2190–2191 (2010).
5. RJ Hardy, SG Thompson, A likelihood approach to meta-analysis with random effects. *Stat. Medicine* **15**, 619–629 (1996).
