# Supplementary material for: metaGE: Investigating genotype x environment interactions through GWAS meta-analysis
Source: PLoS Genet. 2025 Jan 10;21(1):e1011553. doi: 10.1371/journal.pgen.1011553 (PMC11756807; doi:10.1371/journal.pgen.1011553)
Supplement: S2 Text — (PDF) [file pgen.1011553.s002.pdf]

Supporting Information for

metaGE: Investigating genotype x environment interactions through GWAS meta-analysis

Annaïg De Walsche, Alexis Vergne, Renaud Rincent, Fabrice Roux, Stéphane Nicolas, Claude Welcker, Sofiane Mezmouk, Alain Charcosset and Tristan Mary-Huard

Corresponding Author name: Tristan Mary-Huard.
E-mail: tristan.mary-huard@agroparistech.fr

Supporting Information Text 2: Details of the simulation framework.

Phenotypic values simulation procedure. The genotypic data, the experimental design, and the environmental covariates used in the simulation study are all drawn from https://doi.org/10.15454/AEC4BN and https://doi.org/10.15454/IASSTN. On this basis and for each simulation run, phenotypic values were simulated for the 247 available hybrids in the 22 environments of the experiment. Phenotypic values in environment k were generated as a sum of QTLs effects, genetic background effects and an experimental error:

$$Y_k = Q_k + B_k + E_k.$$

We describe the generation of each of these three components in what follows.

QTL effects simulation We defined four different types of multi-environmental QTL effects:

1. **Fixed effects:** The QTL effect is the same across all environments. A value of 1 was assigned to the difference between the effects of alleles "1" and "0" for all environments (i.e. $\beta_{Q_{ik}} = 1$ for $k = 1, ..., 22$).

2. **Completely Random effects:** The QTL effects are completely random across all the environments. The allelic effects associated with each environment were drawn independently from a normal distribution (i.e. $\beta_{Q_{ik}} \sim \mathcal{N}(0, 1)$ for $k = 1, ..., 22$).

3. **Random effects based on the environment correlations QTLs:** QTL effects correlations reflect the correlations between environments. All QTL effects are drawn jointly from an $\mathcal{N}(0_K, \Sigma)$, with the inter-environment correlation matrix Σ built from the environmental covariates (i.e. $\beta_{Q_i} \sim \mathcal{N}(0_K, \Sigma)$). The matrix Σ is computed as $\exp(-\frac{1}{5}D^2)$ where D contains the Euclidean distances between the scaled and centred covariables of each pair of environments. The environmental covariates used are the soil water potential (Psi), the maximum temperature (Tmax), the night temperature (Tnight), the cumulated global radiation (Rad), the leaf to leaf Vapour Pressure Difference (VPDmax) and the Evapotranspiration (ET0).

4. **Fixed effect depending on an environmental covariate QTLs:** QTL effects are generated from a single environmental covariate C centred and scaled (i.e. $\beta_{Q_{ik}} = s_i C_k$ where $s_i \in \{-1, 1\}$ for $k = 1, ..., 22$) corresponds to the (randomly chosen) sign of the relationship. The covariates used were the maximum temperature averaged (Tmax), the night temperature averaged (Tnight), and the soil water potential averaged (Psi).

Each simulation run included 12 QTLs of one type, which were categorized into three minor allele frequency (MAF) groups: low [0.2, 0.25], medium [0.3, 0.35], and high [0.4, 0.45], with four QTLs assigned to each MAF category.The 12 QTL positions were randomly sampled across 8 out of 10 chromosomes, i.e. two specific chromosomes were kept free of QTLs, the remaining ones containing at least one QTL. QTLs were separated by at least 10 Mb.

The global QTL variance $\sigma^2_{Q_k}$ was defined as the variance of the sum of each individual QTL contribution, i.e. $\sigma^2_{Q_k} = V(\sum_{i=1}^{n_{\mathrm{QTL}}} X_{Q_i}\beta_{Q_{ik}})$ where X_{Q_i} is the incidence matrix of QTL i and $\beta_{Q_{ik}}$ is the allelic effect of QTL i in environment k.

Genetic background effects simulation One thousand positions were randomly sampled across the genome to contribute to the genetic background. The effects of the genetic background β_{B_i} were sampled from $\mathcal{N}(0_K, \Sigma)$, thus ensuring higher correlations between genetic values of individuals sharing similar environments. We defined the global genetic background variance $\sigma^2_{B_k}$ as the variance of the sum of each individual marker contribution, i.e. $\sigma^2_{B_k} = V(\sum_{i=1}^{n_{\mathrm{B}}} X_{B_i}\beta_{B_{ik}})$ with X_{B_i} the incidence matrix of marker i and $\beta_{B_{ik}}$ the allelic effect of marker i in environment k. Then, QTLs effects $\beta_{Q_{ik}}$ were re-scaled to ensure a contribution of 44% of the QTL variance $\sigma^2_{Q_k}$ to the total genetic variance $\sigma^2_{G_k} = \sigma^2_{Q_k} + \sigma^2_{B_k}$ in each environment.

Experimental error simulation In each environment k experimental errors were simulated from normal distribution $\mathcal{N}(0, \sigma^2_{E_k})$, with $\sigma^2_{E_k}$ calibrated to achieve a heritability $h^2 = 0.5$. The experimental design matrix was built in a way that ensured an average of 1.3 repetitions per genotype and per environment. Finally, the simulated phenotypic values were defined as $Y_k = X_Q\beta_{Q_k} + X_B\beta_{B_k} + E_k$.

GWAS of simulated phenotypes. The environment-per-environment GWAS analyses were performed using FastLMM (1). For each marker m and for a given environment, the analysis was performed using the following model:

$$Y = \mathbf{1}\mu + X_m\beta_m + Qv + U + E,$$
$$U \sim \mathcal{N}(0, \sigma_G^2 K),\ E \sim \mathcal{N}(0, \sigma_E^2 I) \text{ and } U \perp\!\!\!\perp E,$$

where Y is the vector of the phenotypic observations in the considered environment, μ is the intercept, $\mathbf{1}$ is a vector of ones, X_m is the vector of genotypes at the tested marker m, β_m is the effect associated to the allele 1 of the marker m, Q is a structure incidence matrix, v is the vector of population structure effects, U is a vector of random polygenic effects, σ_G^2 being the residual polygenic variance, K is the matrix of kinship between lines, E is the vector of remaining residual effects with variance σ_E^2, and I is the identity matrix. The structure incidence matrix Q containing the population memberships was obtained from the ADMIXTURE software (2) considering four populations.

References

1. C Lippert, et al., Fast linear mixed models for genome-wide association studies. *Nat. methods* **8** (2011).
2. DH Alexander, J Novembre, K Lange, Fast model-based estimation of ancestry in unrelated individuals. *Genome Res.* (2009).
