# Supplementary material for: metaGE: Investigating genotype x environment interactions through GWAS meta-analysis
Source: PLoS Genet. 2025 Jan 10;21(1):e1011553. doi: 10.1371/journal.pgen.1011553 (PMC11756807; doi:10.1371/journal.pgen.1011553)
Supplement: S3 Text — (PDF) [file pgen.1011553.s003.pdf]

Supporting Information for

metaGE: Investigating genotype x environment interactions through GWAS meta-analysis

Annaïg De Walsche, Alexis Vergne, Renaud Rincent, Fabrice Roux, Stéphane Nicolas, Claude Welcker, Sofiane Mezmouk, Alain Charcosset and Tristan Mary-Huard

Corresponding Author name: Tristan Mary-Huard.
E-mail: tristan.mary-huard@agroparistech.fr

Supporting Information Text 3: Additional analysis of real data applications.

Table S1. Number of QTLs identified by the `metaGE` FE procedure that were found significant in individual analyses of the Arabidopsis dataset.

Number of QTLs	Identified in # Environments
21	1
19	2
11	3
0	≥ 4

Complementary results of the Maize dataset.

Results of the `METAL` and the `mash` procedures. Three different meta-analysis procedures were used to analyze the 22 GWAS summary statistics: the `metaGE` random-effect (RE) procedure, the `mash` procedure using both canonical and data-driven covariance matrices (1) and the `METAL` procedure (2) which does not account for correlations between environments. Fig S2 shows the resulting histograms and Q-Q plots of the p-value distributions from the `METAL` and the `metaGE` random-effect procedures for quality control purposes. The `METAL` q-q plot clearly exhibits an inflated proportion of low p-values, with more than 256,000 p-values under 0.01, compared to the ≈6,000 that one would expect under the full H_0 hypothesis. This inflated proportion results in declaring 40% of the markers as associated after the local score approach correction for multiple testing. In comparison, the `metaGE` RE procedure efficiently controls the type I error rate, as demonstrated by the p-value distribution. It led to the identification of 527 SNPs clustered into 52 regions. The `mash` procedure identified 3,067 SNPs, of which 356 were also detected by the `metaGE` procedure. Notably, the three putative QTLs exhibiting the most significant association peaks in the `metaGE` random-effect analysis, located on chromosomes 3 (*QTL3_120.0*), 6 (*QTL6_20.3*), and 7 (*QTL7_41.4*), were similarly identified by the `mash` procedure (Fig S3A). Moreover, on chromosome 9, the `mash` procedure detected 909 SNPs spanning a broad region from 13.4Mb to 154.4Mb, which were not identified by the `metaGE` procedure. The z-score heatmap of these SNPs revealed relatively moderate effects across the environments (Fig S3B). Most of the significant SNPs on chromosome 9 detected by the `mash` procedure had individual GWAS p-values below 1e-3 in either none (22%) or only one (51%) of the 22 environments.

Night temperature meta-regression tests applied to the Maize dataset. Regarding night temperatures during the grain filling period, hot conditions may affect ovary development and grain growth, carbon translocation or photosynthesis. Interestingly, rapid senescence was observed in heat scenarios as well as smaller individual grain sizes and reduced number of grains per ear in the two environments with extreme conditions (Cra). The QTL2_234 found on chromosome 2 was also identified in platform experiments ((3, 4)) and corresponds to a QTL of plant growth rate in well-watered conditions (pos234) and radiation-interception efficiency (pos233.9).

Complementary results of the EU-NAM Flint dataset.

Results of the `METAL` and `mash` procedures. To analyze the 32 GWAS summary statistics, we applied three different meta-analysis methods: the `metaGE` random-effect (RE) procedure, the `mash` method with both canonical and data-driven covariance matrices (1), and the `METAL` method (2), which does not account for correlations between environments. Fig S5 presents histograms and Q-Q plots of the p-value distributions from both the `METAL` and the `metaGE` RE methods used for quality control. The Q-Q plot from the `METAL` method shows clear inflation of low p-values, with over 1,000 p-values below 0.01—far exceeding the ≈50 expected under the complete null hypothesis (H_0). This inflation leads to more than 30% of markers being declared significant after the FDR correction. In contrast, the random-effect method effectively controls the type I error rate, as indicated by the p-value distribution. This approach identifies 168 SNPs grouped into 16 regions. The `mash` procedure identified 278 SNPs, of which 136 SNPs were also detected by the `metaGE` random-effect procedure. In particular, the three putative QTLs with the most significant association peaks from the `metaGE` RE procedure, located on chromosomes 1 (pos 117.6Mb), 6 (pos 84.2Mb) and 10 (pos 43.4Mb), were also identified by the `mash` procedure (Fig S6).

Application to the Wheat dataset. We consider the Wheat dataset of (5), where GWAS analyses were performed on a panel of 210 wheat lines phenotyped for grain yield in 16 environments (combinations of location x year x treatment). Lines were genotyped at 108,410 SNPs (after quality control) and phenotyped for heading date and grain yield. In (5), the 16 environments were clustered into four groups corresponding to contrasted relationships between heading date and grain yield (low, medium or high correlation between heading date and grain yield, with the last group corresponding to a quadratic relationship between heading date and grain yield). When present, a correlation between heading date and grain yield could be positive or negative, depending on the environment. Both the `metaGE` fixed-effect (FE) and random-effect (RE) procedures were run on the initial per-environment GWAS summary statistics.

The `metaGE` RE procedure identified 15 QTLs (Fig S8A), with each involving one marker except for one QTL located on chromosome 4D that involved three markers. Three of these regions were located at less than 1.6 Mb from known flowering genes or heading date QTLs detected on the same panel in (6). Five of the detected regions colocalized (less than 2Mb) with yield component QTLs detected in (6). For all these colocalizations, the tests were much more significant with the `metaGE` approach than were those from (6) that were based on a standard GWAS model.

In addition, we applied the `mash` procedure using both canonical and data-driven covariance matrices (1) and the `METAL` procedure (2) which does not account for correlations between environments. Fig S9 shows the resulting histograms and Q-Q plots of the p-value distributions from the `METAL` and the `metaGE` RE procedures for quality control purposes. The `METAL` q-q plot clearly exhibits an inflated proportion of low p-values, with more than 21,000 p-values under 0.01, compared to the ≈1,000 that one would expect under the full H_0 hypothesis. This inflated proportion results in declaring more than 8% of the markers as associated after the local score approach multiple testing correction. In comparison, the `metaGE` RE procedure efficiently controls the type I error rate, as demonstrated by the p-value distribution. The `mash` procedure identified 19 SNPs of which 13 SNPs were also detected by the `metaGE` RE procedure (Fig S10).

The `metaGE` FE procedure was also applied to detect markers with stable effects across environments. In total 11 QTLs were identified, highlighting some very significant association peaks, especially on chromosome 1D (involving 16 markers), chromosome 6B (involving 13 markers) and chromosome 7B (involving 19 markers) (Fig S8B). As expected because of the choice of the 16 environments, these stable regions did not colocalize with major flowering genes nor with heading date QTLs from (6). Interestingly, two of the detected regions colocalized with yield component QTLs detected in (6), and again the significance was much higher with the `metaGE` approach. A meta-regression test was performed to detect markers with effects correlated to the correlation between heading date and grain yield (cor_HD_GY). The procedure identified 6 QTLs with two main association peaks on chromosomes 2B and 6A, these regions being illustrated in Fig S8C and Fig S8D. Two of these six regions were close to a known flowering gene (Ppd-B1 on 2B).

References

1. SM Urbut, G Wang, P Carbonetto, M Stephens, Flexible statistical methods for estimating and testing effects in genomic studies with multiple conditions. *Nat. genetics* **51**, 187 (2019).
2. CJ Willer, Y Li, GR Abecasis, Metal: Fast and efficient meta-analysis of genomewide association scans. *Bioinformatics* **26**, 2190–2191 (2010).
3. SA Prado, et al., Phenomics allows identification of genomic regions affecting maize stomatal conductance with conditional effects of water deficit and evaporative demand. *Plant Cell Environ.* **41**, 314–326 (2018).
4. LC Maistriaux, et al., Genetic variability of aquaporin expression in maize: From eqtls to a mite insertion regulating pip2;5 expression. *Plant Physiol.* **196**, 368–384 (2024).
5. P Robert, J Le Gouis, TBC , R Rincent, Combining crop growth modeling with trait-assisted prediction improved the prediction of genotype by environment interactions. *Front. Plant Sci.* **11** (2020).
6. G Touzy, et al., Using environmental clustering to identify specific drought tolerance qtls in bread wheat (t. aestivum l.). *Theor. Appl. Genet.* **132**, 2859–2880 (2019).
