# Supplementary figures and images for: metaGE: Investigating genotype x environment interactions through GWAS meta-analysis
Source: PLoS Genet. 2025 Jan 10;21(1):e1011553. doi: 10.1371/journal.pgen.1011553 (PMC11756807; doi:10.1371/journal.pgen.1011553)

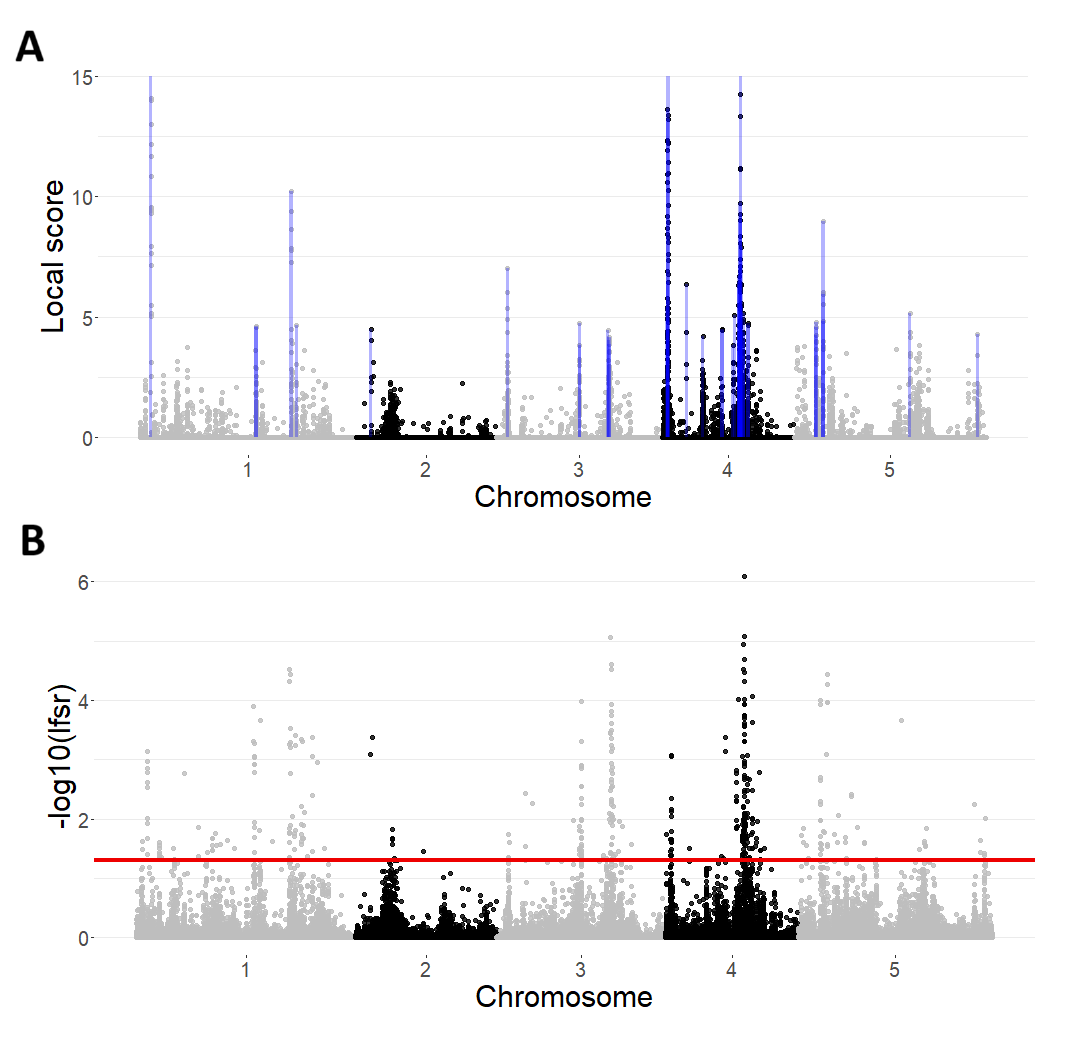

Supplement: S1 Fig — (A) Local scores obtained from the metaGE FE procedure along the chromosomes. The blue boxes represent the significant zones identified. The range of values on the y-axis has been bounded from 0–15 to highlight minor QTLs. (B) Minimum local false sign rate over the environments obtained from the mash procedure along the chromosomes (in log10 scale). The horizontal red line represents the significance threshold of 0.05. (TIF) [file pgen.1011553.s004.tif]

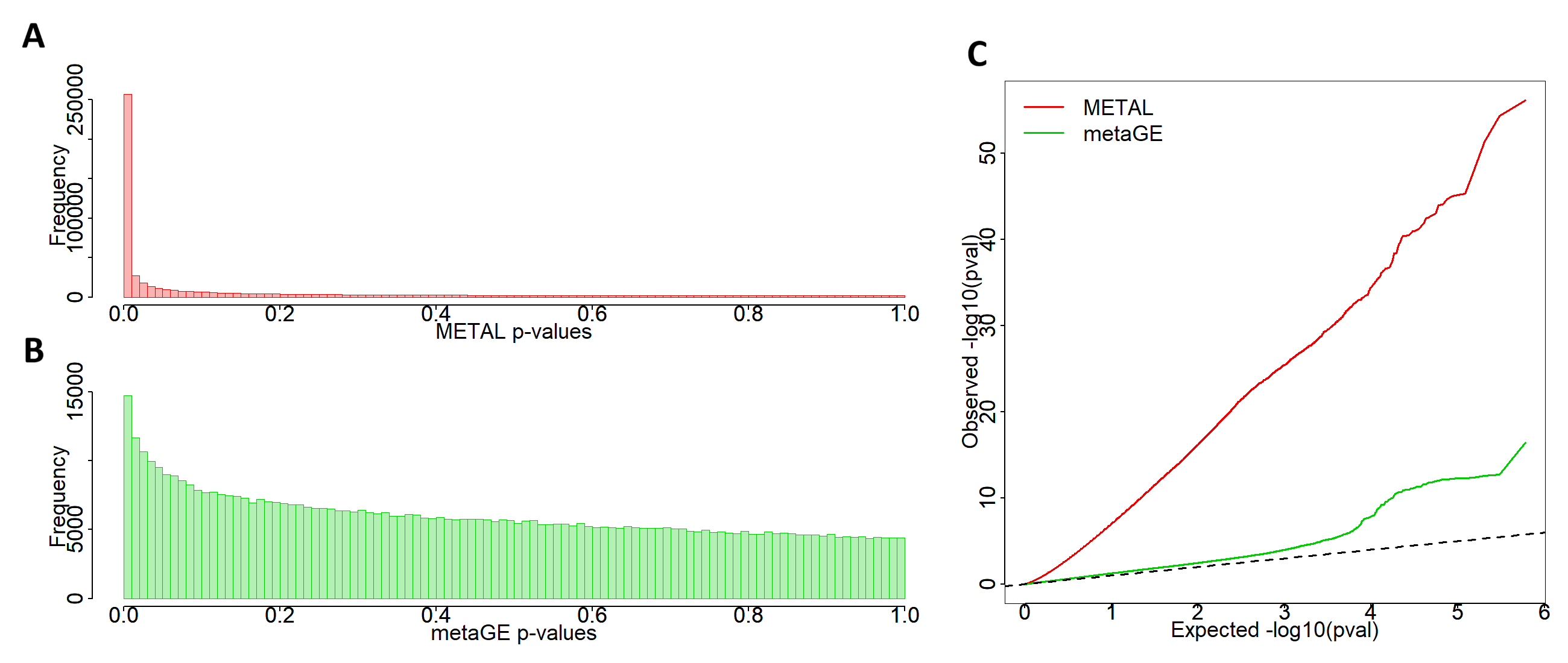

Supplement: S2 Fig — (A) Histogram of the METAL p-values. (B) Histogram of the metaGE RE p-values. (C) QQ-plot of the -log10(p-values) of METAL in red and metaGE in green. The observed -log10(p-values) are compared to the expected quantiles generated by the uniform null distribution. (TIF) [file pgen.1011553.s005.tif]

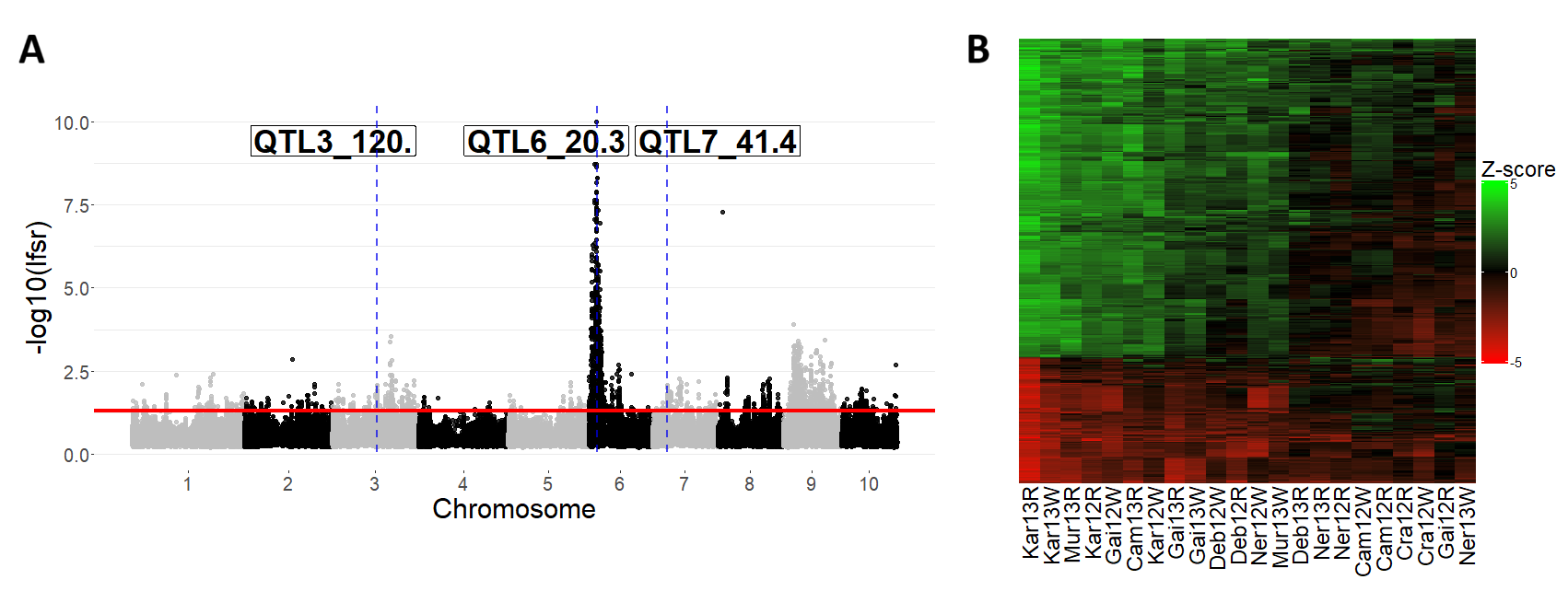

Supplement: S3 Fig — (A) Minimum local false sign rate over the environments obtained from the mash procedure along the chromosomes (in log10 scale). The range of values on the y-axis has been bounded from 0–10 to highlight minor QTLs. The horizontal red line represents the significance threshold of 0.05. The vertical dotted lines correspond to the three main regions identified by the metaGE RE procedure located on chromosomes 3 (QTL3_120.0), 6 (QTL6_20.3) and 7 (QTL7_41.4). (B) Z-scores of the significant SNPs located on chromosome 9, with markers in rows and environments in columns. (TIF) [file pgen.1011553.s006.tif]

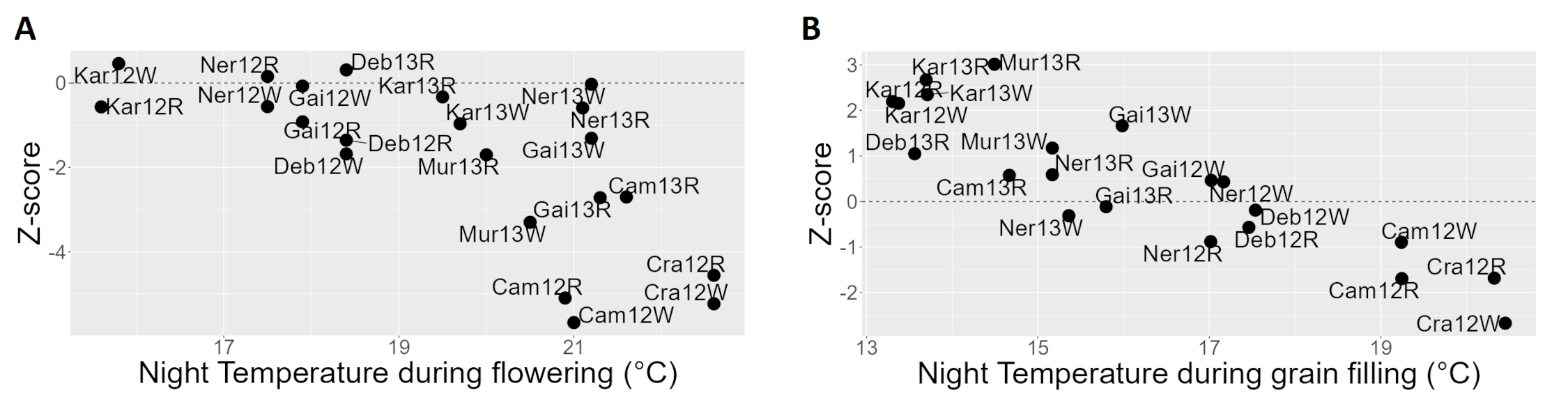

Supplement: S4 Fig — (A) Z-scores as a function of the mean night temperature during the flowering period for the top significant marker detected with the meta-regression procedure (Marker AX-91369217 located on chromosome 6 pos 21Mb). (B) Z-scores as a function of the mean night temperature during the grain filling period for the top significant marker detected with the meta-regression procedure (Marker AX-91123283 located on chromosome 9 pos 28Mb). (TIF) [file pgen.1011553.s007.tif]

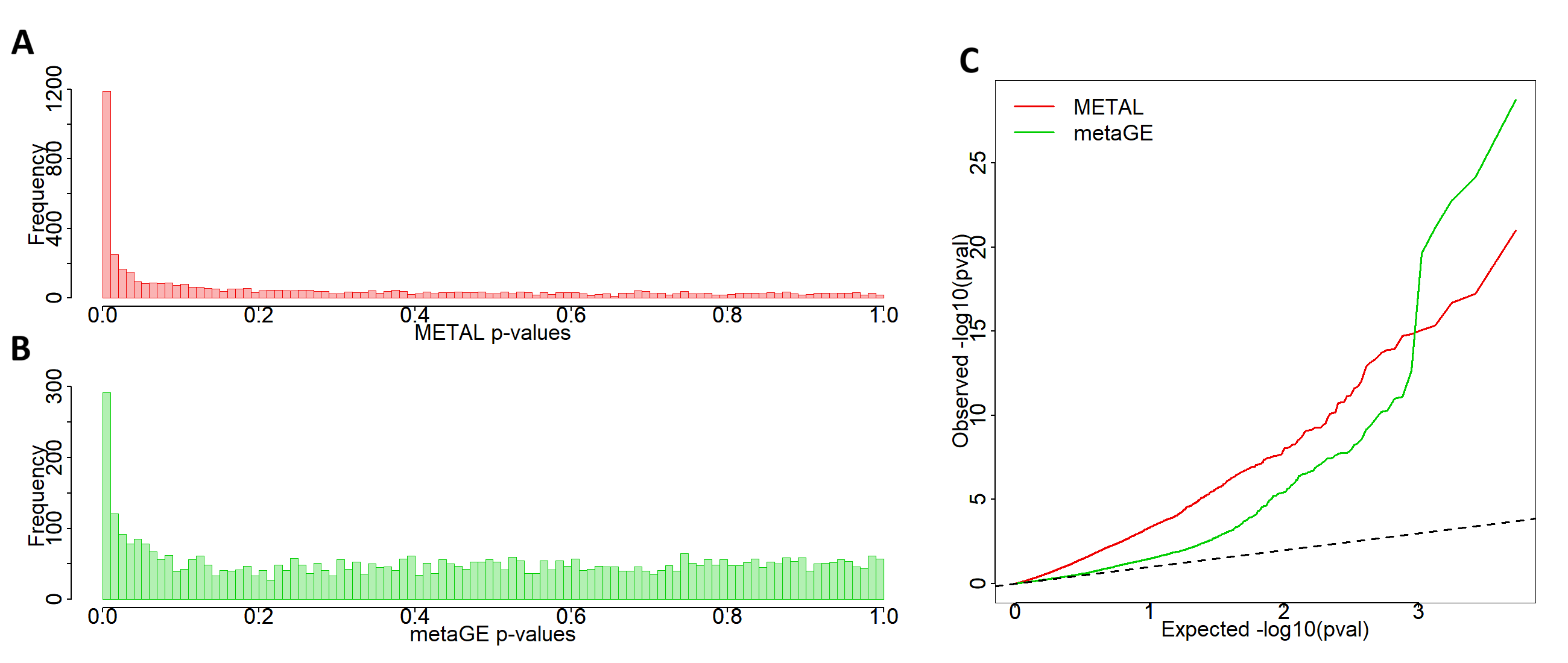

Supplement: S5 Fig — (A) Histogram of the METAL p-values. (B) Histogram of the metaGE RE p-values. (C) QQ-plot of the -log10(p-values) of METAL in red and metaGE in green. The observed -log10(p-values) are compared to the expected quantiles generated by the uniform null distribution. (TIF) [file pgen.1011553.s008.tif]

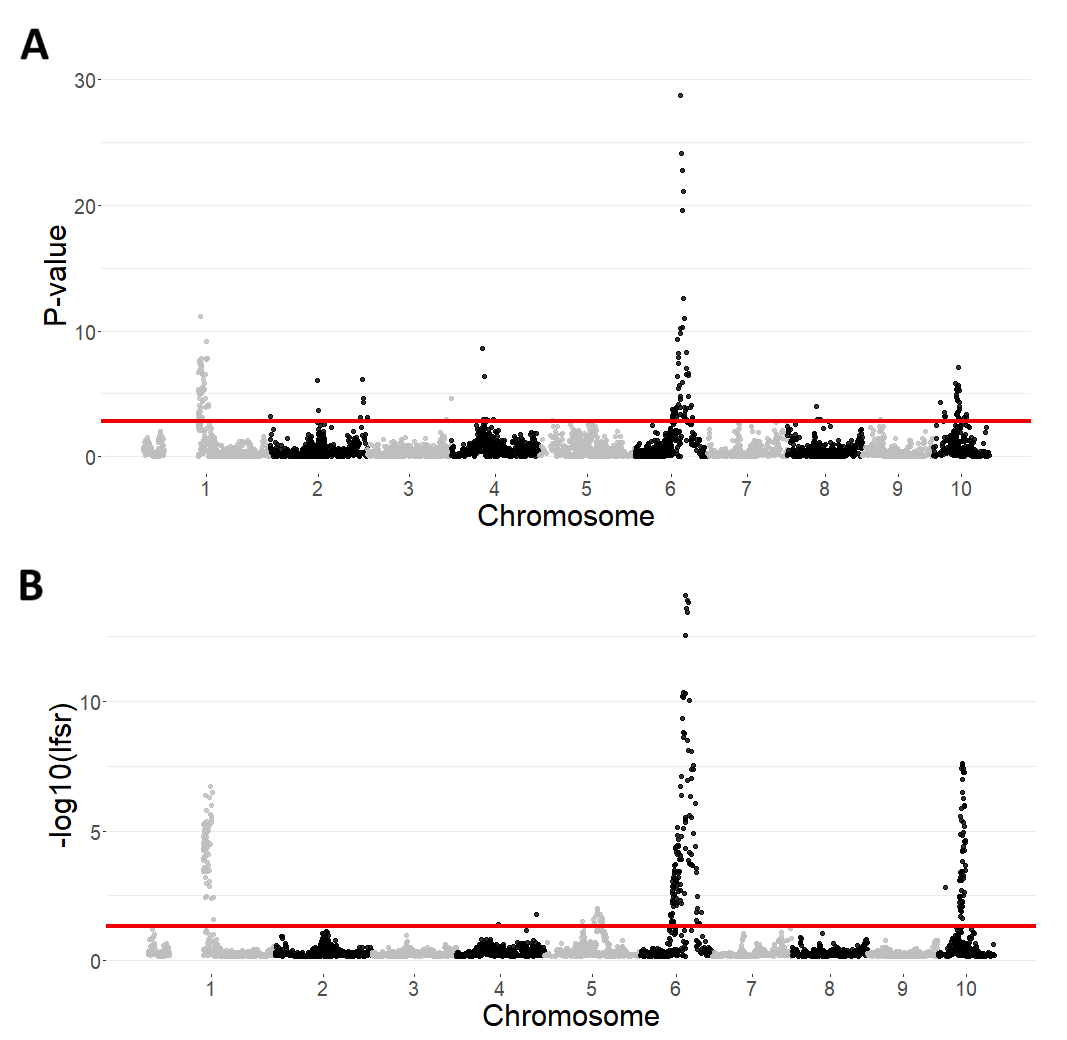

Supplement: S6 Fig — (A) P-values of the random-effect metaGE procedure along the chromosomes (in −log10 scale). The horizontal red line represents the significance threshold of the adaptive Benjamini Hochberg multiple testing correction procedure [46] for a nominal FDR of 0.05. (B) Minimum local false sign rate over the environments obtained from the mash procedure along the chromosomes (in −log10 scale). The horizontal red line represents the significance threshold of 0.05. (TIF) [file pgen.1011553.s009.tif]

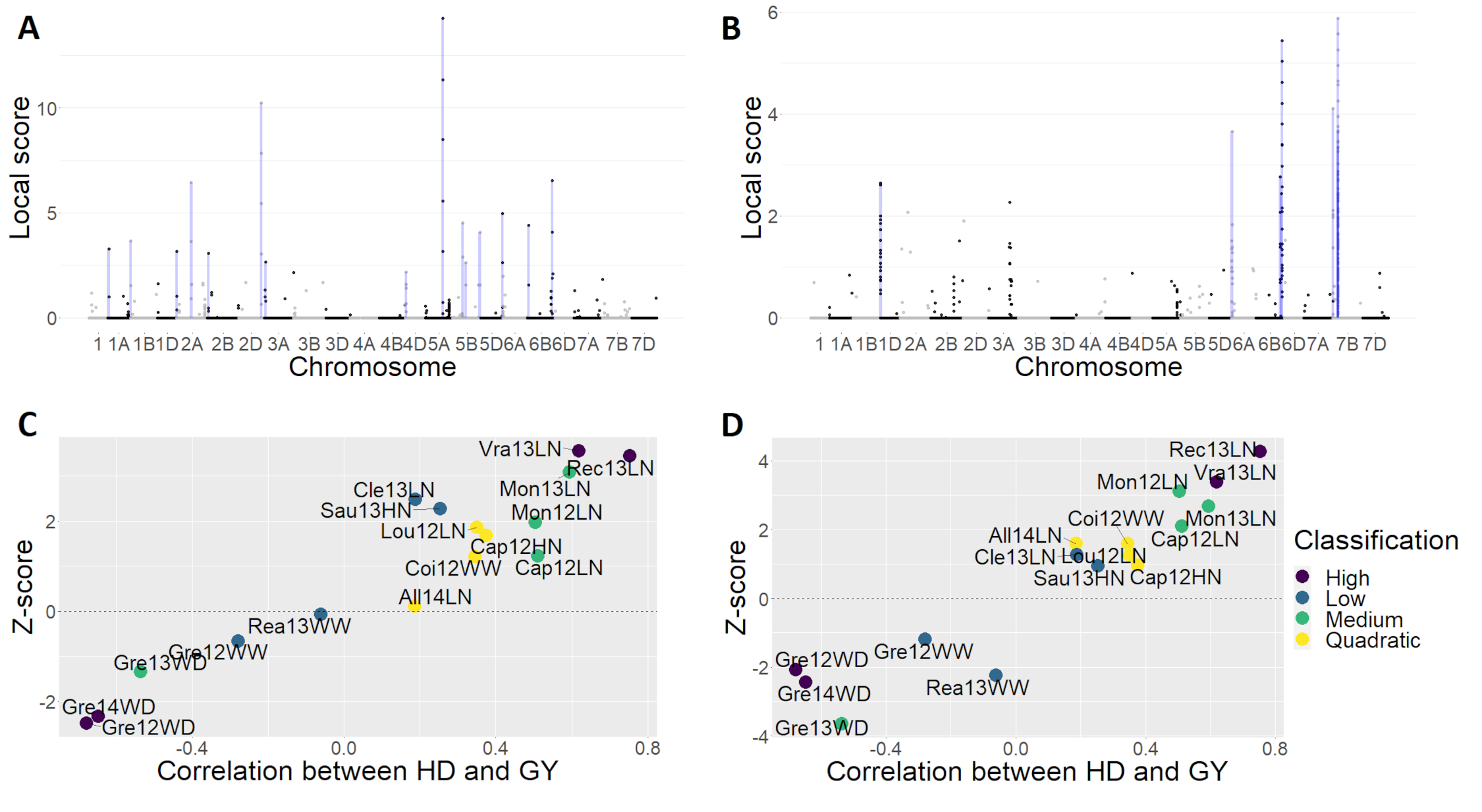

Supplement: S8 Fig — (A) Local score along the chromosomes from the metaGE RE procedure. The boxes represent the significant zones identified. (B) Local score along the chromosomes from the metaGE FE procedure. The boxes represent the significant zones identified. (C) Z-scores as a function of the correlation between the heading date and the grain yield, for the top significant marker detected with the meta-regression procedure (Wheat dataset, marker cfn2941229 located on chromosome 6A). (D) Z-scores as a function of the correlation between the heading date and the grain yield, for the second top significant marker detected with the meta-regression procedure (Wheat dataset, marker cfn1693678 located on chromosome 2B). Colours correspond to the environment classification according to the relationship between heading date and grain yield of [45]. (TIF) [file pgen.1011553.s011.tif]

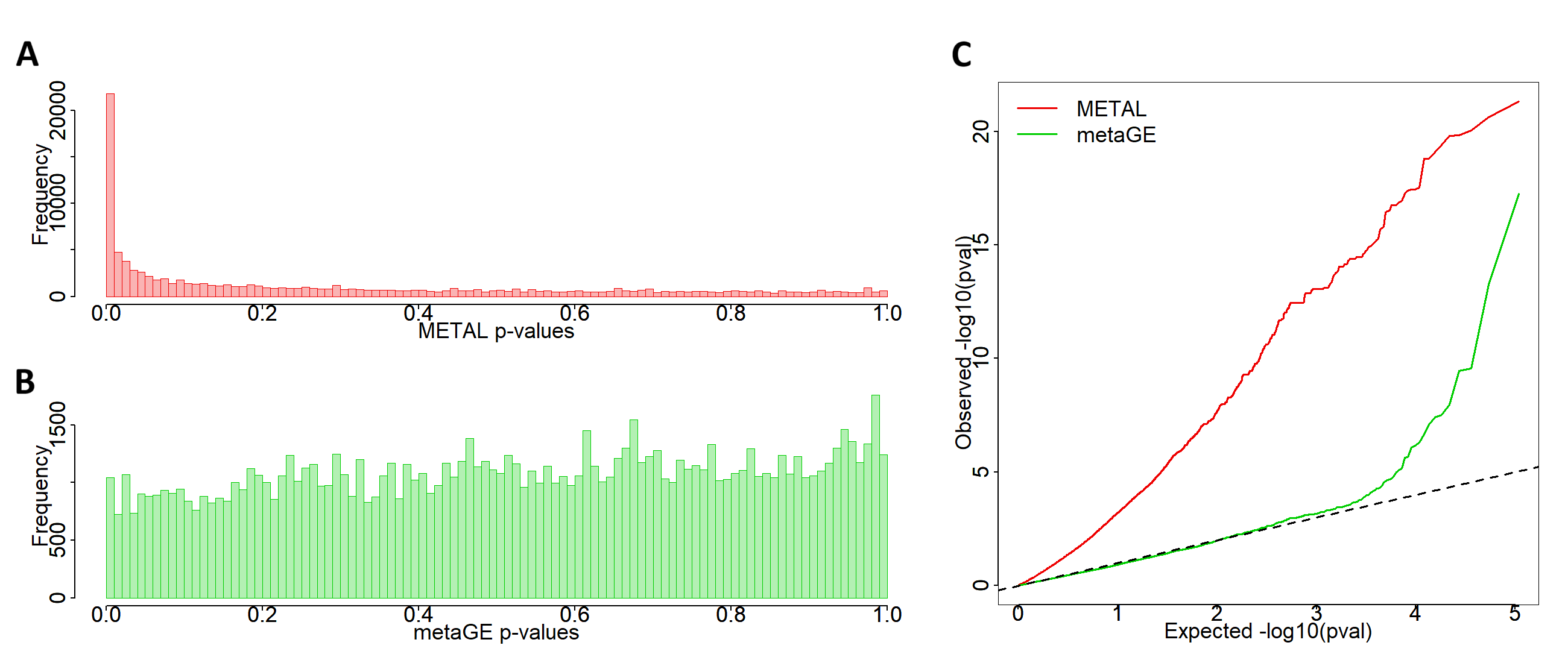

Supplement: S9 Fig — (A) Histogram of the METAL p-values. (B) Histogram of the metaGE RE p-values. (C) QQ-plot of the -log10(p-values) of METAL in red and metaGE RE in green. The observed -log10(p-values) are compared to the expected quantiles generated by the uniform null distribution. (TIF) [file pgen.1011553.s012.tif]

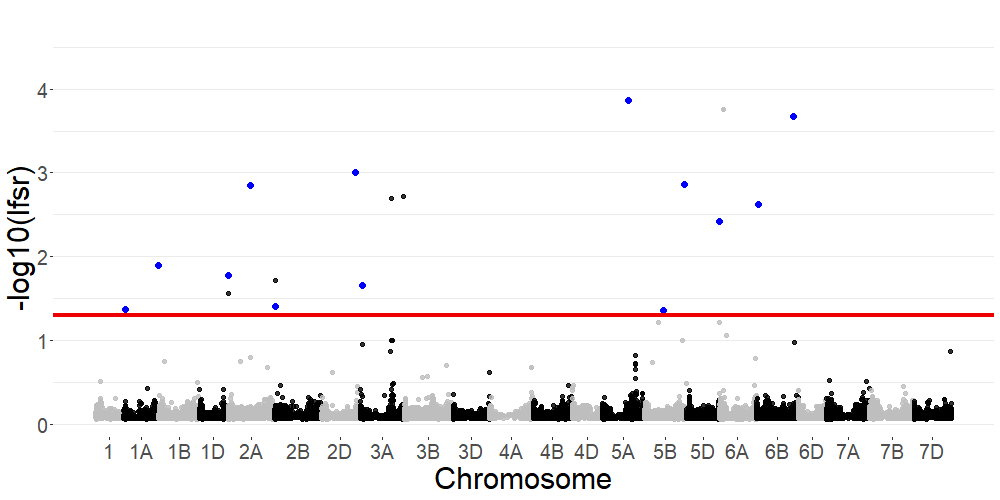

Supplement: S10 Fig — Minimum local false sign rate over the environments obtained from the mash procedure along the chromosomes (in −log10 scale). The horizontal red line represents the significance threshold of 0.05. The blue dots correspond to the SNPs indentified by the metaGE RE procedure. (TIF) [file pgen.1011553.s013.tif]
