# Supplementary material for: metaGE: Investigating genotype x environment interactions through GWAS meta-analysis
Source: PLoS Genet. 2025 Jan 10;21(1):e1011553. doi: 10.1371/journal.pgen.1011553 (PMC11756807; doi:10.1371/journal.pgen.1011553)
Supplement: Attachment — Submitted filename: Response_reviewers_PLOSGenetics.pdf [file pgen.1011553.s014.pdf]

List of revisions and Answer to Reviewers for the paper
metaGE: Investigating genotype x environment interactions through GWAS meta-analysis

We sincerely thank the reviewers for their valuable comments and suggestions, which we incorporated into the revised version of the paper. This document provides our point-by-point replies, explaining how we addressed each of the Reviewers' comments. To ease the reviewing, major revisions are highlighted in blue in the revised article. The original reviews are included in this document in *italic font*.

1 First review

In this manuscript, De Walsche et al. present metaGE, an R package for the analysis of correlated marker effects in GWAS meta-analyses. The manuscript is generally very well-written. The authors rigorously describe the statistical procedures implemented in their tool, and they apply it to several datasets which illustrate its statistical and practical advantages. Remarkably, the metaGE package can be easily downloaded from CRAN, and contains useful help pages (in which example code can be run smoothly) and a vignette. My main comments are related to the simulation studies and the description of empirical studies. The manuscript would be clearer with more controlled simulation studies (see my comment below), a systematic description of analyses (e.g., regarding significance thresholds), and consistent comparisons (e.g., between metaGE and METAL whenever relevant).

Answer: We thank the reviewer for the positive and constructive feedback regarding our manuscript and the `metaGE` package. Below, we address the different points regarding the simulation studies, the description of empirical studies and the systematic comparisons with other tools.

Material and Methods, Simulation framework: How did you choose QTLs in simulations, and why did you choose to simulate different types of QTLs (FE, RE, Correlated RE, proportional to environmental covariate) together in the same simulation? The simulation results would be much easier to interpret (by the authors) and understand (by the readers) if factors impacting QTL detection power were controlled: allele frequency of QTLs, and type of QTL effect. Currently, it's difficult to make sense of the simulation results because of the heterogeneity in marker effects.

Answer: We modified our simulation analysis according to the reviewer request. In the revised version of the manuscript specific simulations are now run on each type of QTL effect separately. Additionally, we stratified the results based on the QTL allele frequencies (low $[0.2, 0.25]$, medium $[0.3, 0.35]$ and high $[0.4, 0.45]$) to assess the impact of frequencies on detection power. The new simulation framework and the simulation results are detailed in the Simulation framework section in the Materials and Methods, and in the Simulation results section in the Results.

Material and Methods: Please add a subsection about the publicly available datasets that you use: species, number of lines and SNPs, number of environments and environmental covariates, phenotypic traits, and significance threshold used for significance in each dataset. This information should be available in the main text.

Answer: In response, we have added a dedicated subsection titled «Application to MET GWAS analysis »in the Materials and Methods section of the manuscript. This subsection provides a comprehensive description of each dataset. This presentation of the datasets is followed by a description of the analysis that was run, including the tuning of the different statistical thresholds used in the analysis.

Results: Why did you not compare metaGE with METAL in the analysis of maize and wheat datasets? I suggest you consistently provide a description of results from METAL, for comparison.

Answer: The `METAL` analysis was initially restricted to the Arabidopsis dataset and illustrated on a single simulation as both applications led to the same conclusion: as `METAL` does not account for correlations between pvalues series it yields significantly inflated FP rate (on simulations) and number of rejected hypotheses (on the Arabidopsis application). As the same behavior was observed for other datasets (not shown so far) we did not initially include these `METAL` results. In response to reviewers 1 and 2 comments, we have re-analyzed the maize, NAM and wheat datasets using both the `METAL` and `mash` procedures. These comparisons are now provided in the corresponding subsections of the manuscript's Results section for the Arabidopsis dataset and in the supplementary materials for the Maize, EU-NAM Flint and Wheat dataset.

Results, Computational efficiency of the method: How do the computational times compare with competing methods like METAL?

Answer: The computational time for `metaGE` is comparable to that of `METAL` and lower to that of `mash`, which is likely due to the more complex modeling of the various effect patterns in `mash`, which increases the computational burden. These comparisons have now been explicitly included in a dedicated section (Computational efficiency) of the Section Results, where we provide detailed computational times for each method across the different datasets analyzed.

L. 68: Does the p-value need to be produced by a specific type of test? Here, does it need to come from a Wald test? Please mention this information in this paragraph.

Answer: We thank the reviewer for this remark. Most meta-analysis methods including `metaGE` do not require p-values from a specific type of test, such as the Wald test. Our method is flexible and can accommodate p-values from various types of statistical tests, as long as they follow a Uniform(0,1) under the null hypothesis. We have clarified this point in the manuscript and mentioned that p-values from different tests are acceptable in lines 76-81.

(Materials and methods)
In GWAS, p-values are typically obtained by testing that no association exists between the marker under consideration and the phenotypic trait using a Wald test procedure. We point out that meta-analysis methods (including the one presented here) do not require the p-values to be obtained from a specific test procedure: it is only assumed that all p-values from the original GWAS tests are uniformly distributed over the (0,1) interval under the null hypothesis.

L. 83: Please specify that Σ_m is a $K \times K$ matrix.

Answer: The dimension of the Σ_m is now specified.

L. 86: Please use a subscript J for the 0-vector.

Answer: In line 92 (previously 86), we assumed that the environments are grouped together (i.e. $J = 1$), meaning that μ_m is a real number rather than a vector. Therefore, we did not use a subscript for the 0-vector in this context. However, if multiple groups of environments are considered (i.e $J > 1$), μ_m is a vector. We have clarified this in the revised manuscript in lines 92-94 for greater clarity.

(Materials and methods, Fixed effect procedure)
If allelic effects are assumed to be stable across all environments (i.e. $J = 1$), the null hypothesis reduces to $H_0 : \{\mu_m = 0\}$ as in the FE procedure described in S1 Text.

L. 131: Was there a typo in the formula for the estimated variance over environments? Shouldn't it be $(1/(K-1)) \times$ crossproduct, instead of $(1/K) \times$ crossproduct $- 1$?

Answer: The formula for the estimated variance provided in line 139 (previously 131) corresponds to the maximum likelihood estimator. Note that when $\Lambda = \Sigma$, the $(1/K) \times$ crossproduct term is an

unbiased estimator of the total variance coefficient of Z_m, i.e. of $\tau + 1$, which is why one needs to further subtract 1 to get the τ estimator.

L. 134: Could you provide a reference for the test with a mixture of chi-squares, as I don't think it's common knowledge in the quantitative genetics community: for example, case 6 in Self, S. G., & Liang, K.-Y. (1987). Asymptotic properties of maximum likelihood estimators and likelihood ratio tests under nonstandard conditions. Journal of the American Statistical Association, 82(398), 605–610. https://doi.org/10.1080/01621459.1987.10478472

Answer: We thank the reviewer for pointing this out and for suggesting a relevant reference, which is now included in line 143 of the revised manuscript.

L. 140-144: I suggest you use upper-case Z and X (as random variables) when describing estimators, not lower-case z and upper-case X, which is a bit confusing – especially since X is assumed to be fixed for the calculation of the SE of $Z_m^T X$ in L.141.

Answer: We agree that using upper-case letters for random variables will make the notation clearer and avoid confusion. In response, we have revised the manuscript to consistently use upper-case Z and X when describing estimators, particularly in lines 150-152 of the revised manuscript (previously lines 140-144).

L. 145-153: What is the justification for using $\xi = 3$? Please provide an explanation, or even a sensitivity analysis, which would be useful since Fariello et al. (ref. 35) recommend epsilon = 1 or 2.

Answer: As mentioned in the original publication by Fariello et al. [2], ξ should be chosen between the average and the maximum of the set of $-log10(p_m)$. On real dataset analyses, the observed average of $-log10(p_m)$ were $\approx$ 0.5 and the maximum of $-log10(p_m)$ ranged 10 to 28 ($\approx$ 0.9 and 10.9 in the simulation studies) so the choice of $\xi = 3$ or 4 is consistent with the recommendations of the initial article. Furthermore, Bonhomme et al. [1] investigated the behavior of the local score in a GWAS context and recommended ξ values higher than 2. This is consistent with our experience, where we observed that low values of ξ lead to inflated numbers of significant peaks (on real data) and inflated FDR (on simulations). We added a sentence in the manuscript about the choice of the value of ξ in lines 161-164.

(Materials and methods, Multiple test control)
In practice, the value of ξ should be selected between the average and the maximum of the set of $-log_{10}$(p-values). In our analyses the observed average $-log_{10}(p_m)$ range from 0.5 to 0.9, and the maximum value from 10 to 19, leading to a value of ξ set to 3 or 4.

L. 157-159: the links https://doi.org/10.15454/AEC4BN and https://doi.org/10.15454/IASSTN were not working at the time of my review.

Answer: We thank the reviewer for bringing this to our attention, unfortunately we could not reproduce the problem: we double-checked the links (`https://doi.org/10.15454/AEC4BN` and `https://doi.org/10.15454/IASSTN`), and could access the webpage content. Is it possible that the data repository were undergoing maintenance at the time of the review ?

L. 190-195: How did you choose the distance thresholds for merging detected QTLs (1 Mb) and the detection window size (5, 10, or 20 Mb)? Do these choices depend on the rate of LD decay in the population under study?

Answer: When considering a 5Mb window size, we observed a clear discrepancy between the FDR computed on the HO chromosomes and the H1 chromosomes, suggesting that in some cases long-range LD could result in significant signals beyond the considered window around the QTL. This was confirmed by the graphical analysis of the simulation results (e.g. Figure 1) and was consistent with the observation that LD can be high in centromeric regions, that may represent a significant part of the chromosome in maize (see e.g. [4]). The use of larger window size allowed us to illustrate this phenomenon.

Table 1: Please add results from METAL, for comparison with metaGE.

Answer: We have analyzed the simulation runs using `METAL` and `mash`, and have now included the corresponding results in Table 1. We thank the reviewer for this suggestion as it illustrate the advantage of using `metaGE` over classical meta-analysis approaches that do not handle FP error rate efficiently.

Table 2: Please add a legend to describe the numbers in this table: metareg.X and QTL.X, total number of runs per test and number of simulated QTL per run.

Answer: We have added a legend to Table 3 (previously Table 2) and to others tables, to describe the terms and numbers presented. Specifically, we now clarify: Each simulation included four QTLs with fixed effects dependent on each of the three covariates. MR_covariate = meta-regression test associated with the covariate ; QTL_covariate = QTL with fixed effect depending on the covariate ; Tnight = the night temperature ; Tmax = the maximum temperature ; Psi = the soil water potential.

L. 293: "supported" or "corroborated" instead of "validated"?
Answer: The word "validated" was replaced with "corroborated" in line 364 of the revised manuscript, as suggested.

L. 428-429: Please elaborate on the different strategies for filtering H1 markers.

Answer: Thank you for raising this important point. We have elaborated on the different strategies for filtering H_1 markers in the revised manuscript (lines 499-505).
(Discussion, Comparison to existing methods)
The `metaGE` procedure used to estimate the correlation matrix between environments aligns with these approaches. It can be related to the inference procedure of the correlation matrix between traits described in [5, 6] but with a different strategy for filtering H_1 markers. In those methods, the filtering is achieved by directly applying thresholds to p-values or z-scores. In contrast, the `metaGE` filtering approach relies on the posterior probabilities of markers being under H_1 as obtained from the procedure detailed in [3].

L. 488-489: How many new regions did you identify?
Answer: Thank you for pointing this out. We have now clarified the number of new regions identified in our analysis in lines 580-581 of the revised manuscript.

(Discussion, Interpreting variability in QTL effects across environmental conditions)
We performed the genome-wide analysis of the Maize dataset with the `metaGE` meta-regression procedure using three covariates: evapotranspiration, mean night temperature during the flowering and grain filling periods. This analysis led to the identification of 14, 20, and 14 new regions, respectively.

L. 493: "In prep." citations are generally not acceptable. Can't you cite other studies to support your claim about these aquaporin genes?

Answer: We have replaced the "In prep." citation with a suitable reference that supports our claim about the aquaporin genes, see line 586 of the revised manuscript.

2 Second review

The work by Walsche et al Investigated GxE in GWAS meta-analysis using simulated data and four case studies of A. thaliana, maize, and also wheat. Authors introduce a powerful R package that run the analysis in a user-friendly way. However, some comments are listed below to improve the manuscript. - Not clear to me the concept of the MA, does the analysis run on all SNPs or only on the significant ones?

Answer: All meta-analysis procedures presented in the manuscript are conducted on all SNPs, not just the significant ones. The procedures aggregate evidence across studies for each SNP, evaluating their effects in all GWAS. This allows for the detection of associations that may not reach significance in individual studies but could become significant through the meta-analysis.

- What are the advantages of the procedure introduced here over other approaches such as "mash" or even the ones implemented in GenStat? Both are used in plant research. This point should be added to

the discussion.

Answer: We thank the reviewer for raising this important point.

First note that GenStat is not an open source software, which represents a limitation on its own. We did not have access to Genstat, however the meta-analysis procedure described in the GenStat documentation website is very similar to the `METAL` procedure that is now included in our comparisons. We strongly believe that the conclusions presented for `METAL` should also apply to GenStat.

The revised version of the manuscript now includes a systematic comparison between the `metaGE`, `METAL` and `mash` procedures, on both simulated and real data. First, the simulation study clearly illustrates that neither `METAL` nor `mash` efficiently handle the correlations between GWAS results, leading to inflated false positive rates. In comparison, the `metaGE` procedure efficiently models correlations between GWAS, allowing a proper FDR control.

Second, the `metaGE` regression approach can detect QTLs with effects that depend on categorial or quantitative environmental covariates, as well as QTLs with random and fixed effects. This makes it particularly suitable for multi-environment studies and for investigating GxE interactions, a crucial aspect in plant genetics research. Although the `mash` procedure can consider various patterns of effects across the GWAS, it cannot incorporate patterns of effects that depend on environmental covariates.

Lastly, `metaGE` is computationally efficient, being more versatile but as fast as `METAL` and significantly faster than `mash` in some applications, as now illustrated in the Computational efficiency paragraph of the Results Section. We have now clarified this point in the paragraph "Comparison to existing methods" of the Discussion, as suggested, to further emphasize the advantages of our approach compared alternative meta-analysis procedures.

- Is the metaGE analysis time consuming like GenStat, when using large genotypic data. That is not clear here but can be a good advantage.

Answer: As mentioned earlier we could not have access to GenStat. The `metaGE` procedure is designed to be computationally efficient and demonstrated comparable time consuming as `METAL`. All datasets could be were analyzed in less than 4 mn. Detailed computational times are now provided for all benchmarked methods across the different datasets analyzed in Table 4.

Some sentences in the results should be moved to the discussion part, for example, lines 234-244: "This can be explained by the fact that these procedures have less targets than the RE procedure, e.g. the FE procedure aims at identifying QTLs with stable allelic effects, i.e. the QTL of type 1 in our simulations." Please, pay attention for this point all over the results.

Answer: Thank you for your suggestion. We have carefully reviewed the results section and moved interpretative sentences to the discussion where necessary.

References

[1] Maxime Bonhomme, Maria Inés Fariello, Hélène Navier, Ahmed Hajri, Yacine Badis, Henri Miteul, Deborah A. Samac, Bernard Dumas, Alain Baranger, Christophe Jacquet, and Marie Laure Pilet-Nayel. A local score approach improves gwas resolution and detects minor qtl: application to medicago truncatula quantitative disease resistance to multiple aphanomyces euteiches isolates. *Heredity*, 123:517–531, 10 2019.

[2] María Inés Fariello, Simon Boitard, Sabine Mercier, David Robelin, Thomas Faraut, Cécile Arnould, Julien Recoquillay, Olivier Bouchez, Gérald Salin, Patrice Dehais, David Gourichon, Sophie Leroux, Frédérique Pitel, Christine Leterrier, and Magali SanCristobal. Accounting for linkage disequilibrium in genome scans for selection without individual genotypes: The local score approach. *Molecular Ecology*, 26:3700–3714, 7 2017.

[3] T. Mary-Huard, S. Das, I. Mukhopadhyay, and S. Robin. Querying multiple sets of p-values, 2021.

[4] Sandra S Negro, Emilie J Millet, Delphine Madur, Cyril Bauland, Valérie Combes, Claude Welcker, François Tardieu, Alain Charcosset, and Stéphane D Nicolas. Genotyping-by-sequencing and snp-arrays are complementary for detecting quantitative trait loci by tagging different haplotypes in association studies. *BMC plant biology*, 19:1–22, 2019.

[5] Debashree Ray and Michael Boehnke. Methods for meta-analysis of multiple traits using gwas summary statistics. *Genetic Epidemiology*, 42:134–145, 3 2018.

[6] Sarah M. Urbut, Gao Wang, Peter Carbonetto, and Matthew Stephens. Flexible statistical methods for estimating and testing effects in genomic studies with multiple conditions. *Nature genetics*, 51:187, 1 2019.
