# Supplementary material for: metaGE: Investigating genotype x environment interactions through GWAS meta-analysis
Source: PLoS Genet. 2025 Jan 10;21(1):e1011553. doi: 10.1371/journal.pgen.1011553 (PMC11756807; doi:10.1371/journal.pgen.1011553)
Supplement: Attachment — Submitted filename: Response_reviewers_metaGE_PLOSGenetics_2-1.pdf [file pgen.1011553.s015.pdf]

List of revisions and Answer to Reviewers for the paper
metaGE: Investigating genotype x environment interactions through GWAS meta-analysis

We sincerely thank the reviewers for their thorough reviews. We modified the manuscript according to their comments. We provide here a point-by-point answer to their comments, explaining how we addressed each of the Reviewers' comments

1 First review

Figures 2 to 5 were missing from this new submission. I assumed that they were the same as those from the previous submission. Please confirm.

Answer: We confirm that these figures are identical to those included in the first submission. We apologize for the missing figures that are now reincorporated into the revised version of the manuscript.

Table 1: please describe power and FDR in the legend. These are worth defining, especially FDR which here refers to the false positive rate, and not the estimated FDR of detected effects (e.g., by the Benjamini-Hochberg procedure).

Answer: We have updated the legend of Table 1 to include definitions of power and FDR. We now explicitly mention that FDR_chr refers to the percentage of simulation runs exhibiting a significant region on the two H_0 chromosomes, while FDR_XMb refers to the percentage of significant local score peaks located outside windows of X Mb centered on QTL positions.

Table 3: Please mention/remind (e.g., in the title) that the MR test is part of metaGE.

Answer: We updated the table title to explicitly mention that the MR test is part of metaGE.

L. 463, Table 4: 'min' instead of 'mn'

Answer: We have corrected 'mn' to 'min' in line 463 as suggested.

L. 528: 'effectively' instead of 'efficiently'

Answer: We have corrected 'effectively' to 'efficiently' in line 463 and subsequently in the Discussion as suggested.

L. 628: Refer to Table 4 at the end of this sentence.

Answer: We have updated line 628 to refer to Table 4.

2 Second review

The authors have addressed all my concerns, and the article now reads more comprehensive. I just want to draw authors' attention to a minor comment that came to my mind regarding Table 4 and the speed of each procedure. How could Mash take 40 minutes to analyze 500 K SNPs over 22 environments for simulated data, but faster with Maiz data with 600 K SNPs and 22 environments? Same for metaGE_RE, not

clear on which factor is time consuming, number of environments or markers. Is there any conclusion whether the speed is related with number of markers or environments? Regarding GenStat, I used 214 K SNPs with 260 accession over few environments, and it took 2-3 days to get the analysis done. metaGE is really powerful.

Answer: Thank you for your positive feedback and for highlighting this important observation regarding the computational times in Table 4. The differences in computational times between the simulated data and the maize data were due to the fact that these analyzes were initially performed on different machines (a server versus a personal computer). This inconsistency resulted in reported times that were not fully comparable. To address this, we have recalculated all computational times on the same machine to ensure a fair comparison between datasets and methods. The results are now consistent, i.e. the Maize data analyzes are now faster when run on 500K rather than 600k.
